# Assessment of the Stylohyoid Complex with Cone Beam Computed Tomography

**DOI:** 10.5812/iranjradiol.4891

**Published:** 2012-12-27

**Authors:** Dilhan İlgüy, Mehmet İlgüy, Erdoğan Fişekçioğlu, Semanur Dölekoğlu

**Affiliations:** 1 Department of Dentomaxillofacial Radiology, Faculty of Dentistry, Yeditepe University, Istanbul, Turkey

**Keywords:** Stylohyoid Complex, Styloid Process, Cone Beam Computed Tomography

## Abstract

**Background:**

Orientation of the stylohyoid complex (SHC) may be important for evaluation of the patient with orofacial pain or dysphagia.

**Objectives:**

Our purpose was to assess the length and angulations of SHC using cone beam computed tomography (CBCT).

**Patients and Methods:**

In this study, 3D images provided by CBCT of 69 patients (36 females, 33 males, age range 15-77 years) were retrospectively evaluated. All CBCT images were performed because of other indications. None of the patients had symptoms of ossified SHC. The length and the thickness of SHC ossification, the anteroposterior angle (APA) and the mediolateral angle (MLA) were measured by maxillofacial radiologists on the anteroposterior, right lateral and left lateral views of CBCT. Student’s t test, Pearson's correlation and Chi-square test tests were used for statistical analysis.

**Results:**

According to the results, the mean length of SHC was 25.3 ± 11.3 mm and the mean thickness of SHC was 4.8 ± 1.8 mm in the study group. The mean APA value of SHCs was 25.6° ± 5.4° and the mean MLA value was 66.4° ± 6.7°. A positive correlation coefficient was found between age and APA (r = 0.335; P < 0.01); between thickness and APA (r = 0.448; P < 0.01) and also between length and thickness was found (r=0.236).

**Conclusion:**

The size and morphology of the SHC can be easily assessed by 3D views provided by CBCT. In CBCT evaluation of the head and neck region, the radiologist should consider SHC according to these variations, which may have clinical importance.

## 1. Background

The stylohyoid complex (SHC) extends from the styloid process (SP) of the temporal bone to the hyoid bone and is located in front of the stylomastoid foramen. The SHC consists of the SP, the stylohyoid ligament (SHL) and the lesser horn of the hyoid bone (1, 2).

The SP can be seen only as a part of the SHC on radiography. The SHL runs from the SP to the lesser horn of the hyoid bone. The SHL cannot be observed on radiographs unless it is ossified (3, 4). Although conventional radiography of the neck or panoramic radiography is reported for evaluation of SP; it cannot be assessed clearly due to superimposition of different bone structures such as the mandible, teeth or the base of the skull (5-9). Since three dimensional computed tomography (3D-CT) can show SHC variations clearly, evaluation of the SP has been made more confidently (8, 10-13). Cone beam computed tomography (CBCT) is a technique that produces 3D digital imaging at reduced cost and less radiation for the patient than conventional CT scans (14).

The orientation of the SHC may be important for evaluation of the patients with orofacial pain or dysphagia. Even though orofacial pain or dysphagia does not present with an elongated ossified SHC, those symptoms may be due to the angulations of the SHC.

## 2. Objectives

The aim of this study is to assess the length and angulations of SHC using CBCT.

## 3. Patients and Methods

The study group consisted of 3D images of 69 patients in the age range of 15-77 years. In this study, 3D images acquired with Iluma CBCT scanner (Imtec Corporation, Germany) were evaluated retrospectively. All 3D CBCT images of patients who were referred to our radiology department in 2011, were requested because of other pathologies (cyst, tumor, jaw fracture, dental implant analyze and embedded teeth). Digital images were obtained at 120 kVp, 3.8 mA, and a voxel size of 0.2 mm, with an exposure time of 40 seconds. 3D reconstructions were created by reformatting the axial CBCT scans on a local workstation using the Iluma dental imaging software in accordance with the manufacturer's instructions.

Patients with fracture or pathology in the region of the SP were not included in the study. None of the patients had symptoms of ossified SHC. Scans were performed in the axial plane with 2 mm slice thickness. A specialist in oral radiology evaluated the images in a quite darkened room with dual monitors (HP LP2475W, resolution 1920 x 1200). One viewing session was limited to 30 minutes. Care was taken to ensure that 24 hours elapsed between the specialist’s viewing sessions. The images were evaluated by the same observer for a second time after two weeks.

All measurements were made with anteroposterior, right lateral and left lateral views in the 3D interactive module to the end point of ossification.

**Figure 1:**
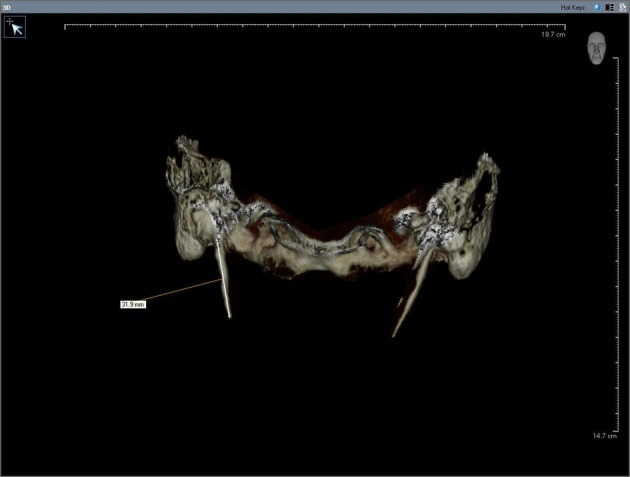
The length of the two segmented ossified SHC on CBCT

The morphology of SHC was recorded to evaluate the general structural appearance, and the number of segments was assessed. The length was defined as the distance between the base of the SP and the tip of the ossified SHC on the anteroposterior view (Figure 1). If there was a segmental ossification of the SL, the distance was measured including the non-ossified parts.

**Figure 2:**
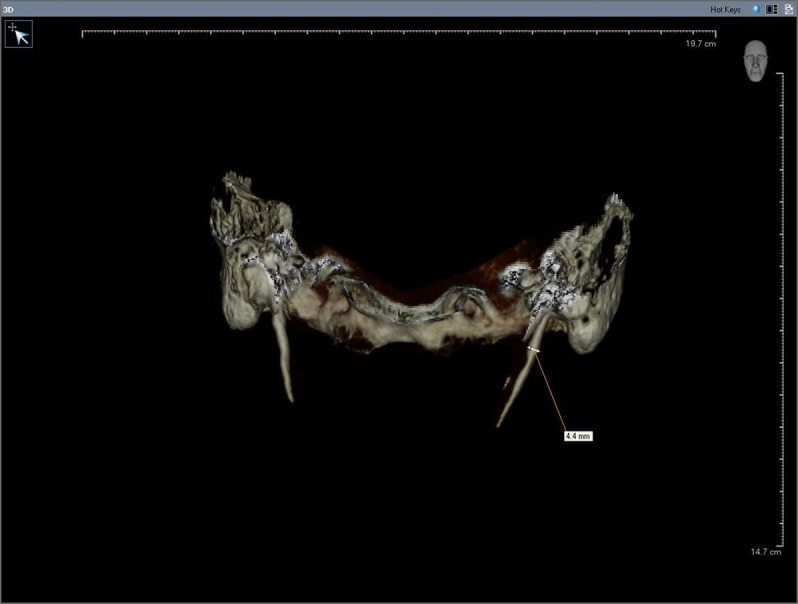
The maximum thickness of SHC ossification on CBCT

**Figure 3:**
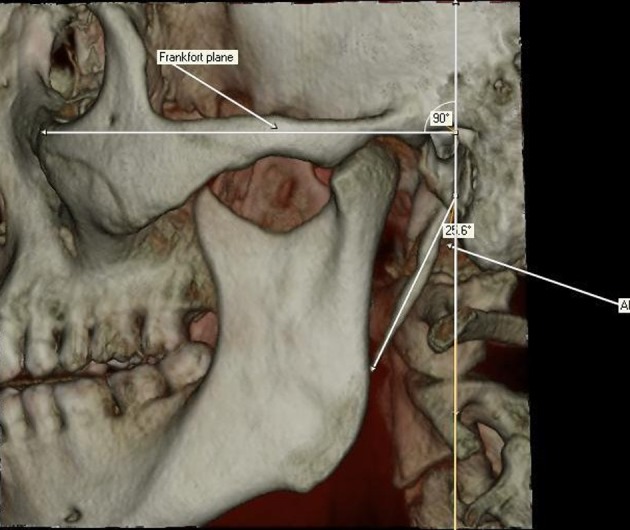
The anteroposterior angle (APA) according to Frankfort plane

The thickness of SHC ossification (Figure 2) was defined as the distance where maximum thickness was seen. The anteroposterior angle (APA) was defined as the vertical line passing from the cranial base of the process, which was vertical to the Frankfort plane (a line passing horizontally from the superior border of the external auditory meatus to the inferior border of the orbital rim) on the lateral view. The angle between this vertical line and the body of the process was measured (Figure 3).

**Figure 4:**
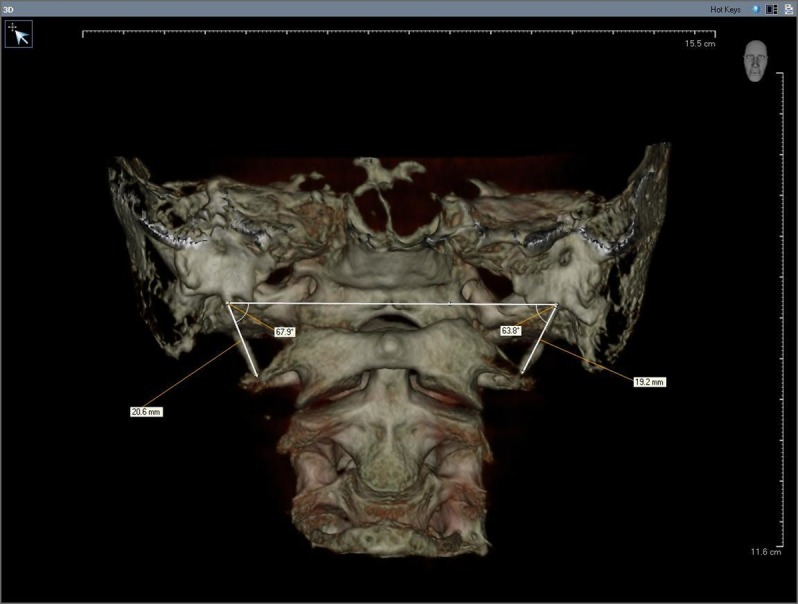
The mediolateral angle (MLA), the angle of intersection of the line connecting both bases of the SP and the longitudinal axis SHC

The mediolateral angle (MLA) was determined as the angle of intersection of the line connecting both bases of the SP and the longitudinal axis of the SHC on the anteroposterior view (Figure 4).

SPSS (Statistical Package for Social Sciences) 15.0 for Windows (SPSS Inc., Chicago, Illinois, USA) was used for statistical analysis. Apart from descriptive statistics, for comparison of continuous variables between different groups, first normality of the data was assessed using Kolmogorov-Smirnov test. When the data distribution was normal, the comparison was performed by Student’s t test and the correlation between the variables was evaluated by Pearson’s correlation coefficient. The qualitative data were evaluated using Chi-square test. Significance was accepted at P < 0.05 level. Intraobserver agreement was calculated using intraclass correlation coefficients (ICCs).

## 4. Results

Of the 69 patients, 36 were female and 33 were male (mean age, 44.30 ± 17.56 years). Totally, 138 SHCs were measured. The results of ICC reflect the reliability of the observer with inter-ratings, which explains that the observer gave similar ratings within the repeated observations for each measurement. Table 1 shows the ICCs for two measurement sessions of length, thickness, APA and MLA. For all re-measurements the observer gave similar ratings (P < 0.01).

**Table 1:** ICCs for Two Measurement Sessions of Length, Thickness, APA and MLA

		ICC	%95 CI	P Value
Length	Right	0.998	0.994-0.999	0.001**
	Left	0.996	0.992-0.998	0.001**
Thickness	Right	0.991	0.980-0.996	0.001**
	Left	0.989	0.976-0.995	0.001**
APA	Right	0.992	0.981-0.997	0.001**
	Left	0.989	0.975-0.995	0.001**
MLA	Right	0.986	0.967-0.994	0.001**
	Left	0.992	0.981-0.997	0.001**

Abbreviations: ICC, Intraclass correlation coefficient; CI, Confidence interval; APA, Anteroposterior angle; MLA, Mediolateral angle

^**^p<0.01

The results of the study showed that there was no statistically significant difference in SHC between different age groups (P > 0.05) as well as between right and left sides.

According to the results, the mean length of SHC was 25.3 ± 11.3 mm and the mean thickness of SHC was 4.8 ± 1.8 mm. The mean APA value of SHCs was 25.6° ± 5.4° and the mean MLA value was 66.4° ± 6.7°.

**Figure 5:**
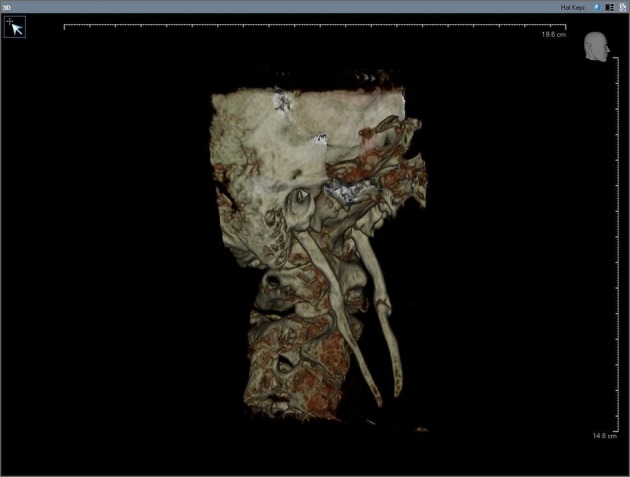
Complete ossification of SHC

On the right side, single segment ossification was found in 28 SHCs (40.6%), two segment ossification was found in 40 SHCs (58%) and three segment ossification was found in one SHC (1.4%). On the left side, single segment ossification was found in 31 SHCs (44.9%), two segment ossification was found in 34 SHCs (49.3%) and three segment ossification was found in four SHCs (5.8%). Only four SHCs were observed as complete ossification (Figure 5).

According to Pearson’s correlation test, there was no correlation between the age of the patients, and length, thickness and MLA values (P > 0.05). A positive correlation was found between age and APA (r = 0.335; P < 0.01). There was a positive correlation between thickness and APA (r = 0.448; P < 0.01) suggesting that the thicker SHC was directed more anteriorly and also a positive correlation between length and thickness was found (r = 0.236, P < 0.01).

Table 2 shows the mean measurements of SHCs according to gender. The mean length of SHC was 28.2 mm for males and 22.5 mm for females. There was a statistically significant difference in SHC length between genders; males had longer stylohyoid processes (P < 0.05). Also the mean values of APA of females were statistically higher than men and the thickness values of the men were statistically higher than women (P < 0.01).

**Table 2:** The Mean Results of the Length, Thickness, APA and MLA Regarding Gender

	Female	Male	P Value
	Mean±SD	Mean±SD	
Length (mm)	22.57±6.02	28.21±14.36	0.042*
Thickness (mm)	4.30±1.81	5.38±1.31	0.006**
APA°	27.18±4.44	23.8±5.12	0.004**
MLA°	65.59±5.48	67.35±6.62	0.230

Student t test

^*^p<0.05

^**^p<0.01

Table 3 shows percentile values for length, thickness, MLA and APA of the SHCs which were analyzed for both sides. The 25-75th percentiles were accepted as the normal group.

**Table 3:** The Percentiles of the Length, Thickness, APA and MLA

	25^th^ Percentile	75^th^ Percentile
**Length (mm)**	19	28.85
**Thickness (mm)**	3.35	6.20
**APA°**	28.95	22
**MLA°**	63.67	69.75

Abbreviations: APA, Anteroposterior angle; MLA, Mediolateral angle

## 5. Discussion

CBCT allows the creation of images in “real time” not only in the axial plane, but also two dimensional images in the coronal, sagittal and even oblique or curved image planes — a process referred to as multiplanar reformation. In addition, CBCT data are amenable to reformation in a volume, rather than a slice, providing 3D information (15). Studies have suggested that CBCT provides accurate and reliable linear measurements for reconstruction and imaging of dental and maxillofacial structures (16-18). Ramadan et al. (19) reported that the percentage of single segment ossification was 75.5%; two or three-segment ossification was 24.5%; complete ossification of the SHC was found in four SHCs. In the present study, on the right side; single segment ossification was found in 40.6%, two segments was found in 58% and three segments was detected in 1.4%. On the left side; single segment ossification was 44.9%, two segments were 49.3% and three segments was 5.8%. Similarly four SHCs were observed as complete ossification. The percentage of single segment was lower and two or three segments was higher than reported in the study performed by Ramadan et al. (19). This may be due to the number of patients in the study groups.

The length of the ossified SHC varies individually. Previous studies reported different values about the normal length of the ossified SHC (3, 5, 20, 21). Some studies with conventional methods reported the normal length of SP as 25-30mm (3, 5, 6, 20-23). Previous studies with panoramic radiographs stated that there was a difference in calcification of the stylohyoid process between genders (24-26). Some authors stated that there was no gender predilection for differences in length (13, 19, 23, 27) which is similar to the results of the present study. Koşar et al. (28) stated that no statistical difference was found between the right and left sides in terms of the length of SP. In the present study, all measurements including length showed no correlation with the side of assessment.

Based on a study conducted by Ramadan et al. (19), instead of mean values for the length of SP, the 25-75th percentile should be accepted as the normal range because of the frequent variations in the normal population. In the previous studies with 3D-CT, the normal range of the length of SHC was reported as 20-40 mm and 21-30 mm (11, 12). In the present study, the normal range was found as 19-28 mm and the mean length was 22.25 mm. Lengths above this range were accepted as “elongated”. The upper limit is not consistent with the study which reported 40 mm as the upper limit (11).

Elongated SP may cause symptoms like a dull pain localized in either or both sides of the throat, with or without referred pain to the ear and the mastoid region on the affected side or cervicofacial pain (13, 29). The patients with Eagle syndrome which is caused by an elongated ossified styloid process may complain of pain on swallowing (odynophagia) or an abnormal sensation of a foreign body in the pharynx (globus hystericus). Other symptoms that aid in the diagnosis include pain with rotation of the head, recurrent headache, vertigo, facial pain, otalgia and cephalalgia (30, 31). The pressure effect of the elongated SP may result in contraction of the surrounding soft tissues. It seems that the length is not enough to explain these complaints (32). Thus, other morphological characteristics of the SHC, such as the angle degree, are necessary to explain the causes (19, 33). During assessment of the ossified SHC, angles are new parameters to clarify the direction. Alterations in the APA or MLA of the ossified SHC may provoke these symptoms. These angles can be easily evaluated by 3D-CT (19). The value of APA varies according to previous studies due to the usage of different measurement methods; while Ramadan (19) reported the APA value as 63.6°, Onbas et al. (10) found it as 93.5°. Yavuz et al. (23) stated 21.4° for the right and 18.5° for the left side. McRae’s and Chamberlain’s lines were used in the first and the latter reports for APA. In our study, the mean APA value of SHCs was found as 25.66° for the right and 25.46° for the left side, which is similar with the control group of the study by Yavuz et al. (23). In the present study the angle between the vertical line and the body of the process was measured for APA, thus the values were smaller than the APAs of the first study in which the horizontal angle was used. Frankfort plane was used as the reference plane in the study similar to the study by Yavuz et al. (23), as it was not easy to visualize the lips of foramen magnum which is necessary for McRae’s or Chamberlain’s lines on 3D views of each subject.

Changes in the APA angle may cause posterior direction of the ossified SHC; therefore, the IX-XIIth cranial nerves, internal carotid artery and internal jugular vein may be compressed between the ossified SHC and lateral mass of the atlas (10, 33).

Previous studies reported the MLA ranging between 67.5° and 72.7° (10, 11, 24, 32). According to our results, the mean MLA was found as 66.4° which is similar to these values. Considering the 25th-75th percentiles as the normal range, Basekim et al. (11) reported the normal range as 65°-75° and Ramadan (19) as 67°-76°. In the present study, this range was found as 63°-69°. Decreased angles may cause close contact between the SHC and the internal carotid artery, while increased angles may cause a compression on the external carotid artery (12).

The size and morphology of the SHC can be easily assessed by 3D views with CBCT. During CBCT evaluation of the head and neck region, SHC should be considered according to these variations by the radiologist, which may be related with clinical symptoms.
